# A comparative review of estimates of the proportion unchanged genes and the false discovery rate

**DOI:** 10.1186/1471-2105-6-199

**Published:** 2005-08-08

**Authors:** Per Broberg

**Affiliations:** 1 Biological Sciences, AstraZeneca R&D Lund, S-221 87 Lund, Sweden

## Abstract

**Background:**

In the analysis of microarray data one generally produces a vector of *p*-values that for each gene give the likelihood of obtaining equally strong evidence of change by pure chance. The distribution of these *p*-values is a mixture of two components corresponding to the changed genes and the unchanged ones. The focus of this article is how to estimate the proportion unchanged and the false discovery rate (FDR) and how to make inferences based on these concepts. Six published methods for estimating the proportion unchanged genes are reviewed, two alternatives are presented, and all are tested on both simulated and real data. All estimates but one make do without any parametric assumptions concerning the distributions of the *p*-values. Furthermore, the estimation and use of the FDR and the closely related q-value is illustrated with examples. Five published estimates of the FDR and one new are presented and tested. Implementations in R code are available.

**Results:**

A simulation model based on the distribution of real microarray data plus two real data sets were used to assess the methods. The proposed alternative methods for estimating the proportion unchanged fared very well, and gave evidence of low bias and very low variance. Different methods perform well depending upon whether there are few or many regulated genes. Furthermore, the methods for estimating FDR showed a varying performance, and were sometimes misleading. The new method had a very low error.

**Conclusion:**

The concept of the q-value or false discovery rate is useful in practical research, despite some theoretical and practical shortcomings. However, it seems possible to challenge the performance of the published methods, and there is likely scope for further developing the estimates of the FDR. The new methods provide the scientist with more options to choose a suitable method for any particular experiment. The article advocates the use of the conjoint information regarding false positive and negative rates as well as the proportion unchanged when identifying changed genes.

## Background

The microarray technology permits the simultaneous measurement of the transcription of thousands of genes. The analysis of such data has however turned out to be quite a challenge. In drug discovery, one would like to know what genes are involved in certain pathological processes, or what genes are affected by the intervention of a particular compound. A more basic question is 'How many genes are affected or changed?' It turns out that the answer to this basic question has a bearing on the other questions.

### The proportion unchanged

In the two-component model for the distribution of the test statistic the mixing parameter *p*_0_, which represents the proportion unchanged genes, is not estimable without strong distributional assumptions, see [1]. Assuming this model the probability density function (pdf) *f*^*t *^of a test statistic *t *may be written as the weighted sum of the null distribution pdf  and the alternative distribution pdf 



If, on the other hand, we know the value of *p*_0 _we can estimate  e.g. through a bootstrap procedure as described in [1], and thus obtain also .

The mixing parameter *p*_0 _has attracted a lot of interest lately. Indeed it is interesting for a number of applications. Here follow four examples.

1) Knowing the proportion changed genes in a microarray experiment is of interest in its own right. It gives an important global measure of the extent of the changes studied.

2) The next example concerns FDR. Suppose we reject null hypothesis *j*, and call gene *j *significantly regulated, when the corresponding *p*-value *p*_*j *_falls below some cutpoint *α*. The question that motivates the FDR concept, which originates from [2], is: "What proportion of false positives is expected among the selected genes?" A goal would then be to quantify this proportion, and one possible estimate is



where '^' above a quantity (here and henceforth) means that it is a parameter estimate, *P*_(*L*) _is the largest *p*-value not exceeding *α *and *p*(*α*) is the proportion significantly regulated genes which equals the proportion of the *p*-values not exceeding *α*, see also [3]. In practice *P*_(*L*) _will be very close to *α*, and may be replaced by the latter. We thus obtain an estimate of *p*_0 _× *α*/*p*(*α*), which verbally translates into "the number of true null cases (*Np*_0_) multiplied by their probability of ending up on the top list (*α*) divided by the number of selected cases (*Np*(*α*))". Putting *p*_0 _≡ 1 above and rejecting hypotheses *j *with the estimated *FDR*(*p*_*j*_) less than or equal to *β*, will give a test procedure controlling the *FDR *at the level *β*, i.e. we may expect that the FDR is no more than *β *[2-4]. By finding good estimates of *p*_0 _(and *FDR*) we may increase the power to detect more true positives at a given FDR bound.

3) Knowing *p*_0_, we may calculate the posterior probability of a gene being a Differentially Expressed Gene (a DEG) as



see [1]. Also, (3) equals one minus the local false discovery rate: 1 - *p*_1_(*x*) = *LFDR*(*x*) = *p*_0 _*f*^*t*^_0_(*x*) / *f*^*t*^(*x*) [1].

4) Knowing *p*_0_, it is also possible to estimate the number of false positives and false negatives at a given cutpoint *α *as a proportion of the total number of genes. Call these proportions the false-positive and false-negative rates, and denote them by *FP*(*α*) and *FN*(*α*), respectively. In the samroc methodology [5] one calculates estimates of these quantities as



and



One may choose a *p*-value threshold *α*_*min*_, which minimises the amount of errors *FP*(*α*) + *FN*(*α*). Alternatively, one may want to fine-tune the test statistic such that it will minimise the errors at a given threshold. Or, one may try to do both, as suggested in [5], see also [6].

Earlier research providing estimates of *p*_0 _include [1,3,7-13]. Articles that compare methods for estimating *p*_0 _and *FDR *include [12,14]. I will focus on the FDR as the main use of *p*_0_.

In this article, the formulation of the theory is in terms of *p*-values rather than in terms of test statistics. Two basic assumptions are made concerning their distribution. First, it is assumed that test statistics corresponding to true null hypotheses will generate *p*-values that follow a uniform distribution on the unit interval, e.g. [15]. Thus, under the null distribution, the probability that a *p*-value falls below some cutpoint *α *equals *α*. Second, *p*-values are, unless stated otherwise, assumed to be independent. Empirical investigations will assess the effects of deviations from the second assumption.

The use of *p*-values means lumping up- and downregulated genes together. However, one may look separately at the two tails of the distribution of the test statistic to assess differential expression corresponding to up- and downregulation.

This article will not concern how *p*-values are calculated, but rather how they are used to calculate estimates of *p*_0 _and *FDR*, and draw conclusions based on this evidence. It is assumed that *p*-values capture the essence of the research problem. Neither does the article treat the choice of an optimal test statistic. For illustration the *t*-test will be used repeatedly without regard to whether there are better methods or not. By the *t*-test we mean the unequal variance *t*-test:  for sample means *mean*_1 _and *mean*_2_, sample variances , , and sample sizes *n*_1 _and *n*_2_. We apply the *t*-test to simulated normally distributed data and a permutation *t*-test to real data, where normality may be uncertain. All calculations were performed in R. The methods presented are available within packages for the free statistical software R [16,17] and take a vector of *p*-values as input and output an estimate of *p*_0 _and of *FDR*. Emphasis lies on methods available within R packages downloadable from CRAN [18] or Bioconductor [19]. Inevitably any review will exclude interesting work, but time and space limitations will not permit an all comprehensive review. The new and highly interesting concept of a local false discovery rate (LFDR) [1] only receives a cursory treatment.

This article builds on and finds motivation from the experience of the analysis of microarrays, which typically assay the expression of 10,000 or more genes. However, the methods presented apply equally well to other high dimensional technologies, such as fMRI or Mass Spectrometry.

### False discovery rate

In the analysis of microarray experiments, the traditional multiple test procedures are often considered too stringent, e.g. [20] and [3]. In the last decade alternatives based on the concept of an FDR have emerged. For more details consult e.g. [2,3,9,11,21,22]. There are different definitions proposed, but loosely speaking one would want to measure the proportion of false positive genes among those selected or significant. Loosely put the *FDR *may be interpreted as the proportion of false positives among those genes judged significantly regulated. Equation (2) is the *FDR *estimate presented in [3].

Denote by *E*[*X*] the expectation (or true mean) of any random variable *X*. With *V *the number of false positives given a certain cut-off and *R *the number of rejected null hypotheses, one may define the *FDR *as the expectation of the ratio of these quantities, or

,

where care is taken to avoid division by zero.

In [10] and [11] the FDR is estimated as the ratio of the expected proportion of false positives given the cut-off to the expected proportion selected. Viewed as a function of a cut-off *α*, such that genes g_i _with *p*_*i *_less than *α *are judged significant in terms of *p*-values, following the continuous cumulative distribution function (cdf) *F*, the *FDR *estimate is



which is nearly equal to (2) with the exception that the *P*_(*L*) _has been replaced by its upper bound, and the step-wise empirical distribution by a smooth version, either a parametric model or a smoothed version of the empirical distribution. Thus, the *FDR *is now a continuous function instead of piece-wise continuous with jumps at each observed *p*-value. This ratio of expected values tries to estimate the expectation of a ratio: In general such an approach will give an overestimation, but in practice this will have little effect, see the Additional file.

The related concept of the positive *FDR*, *pFDR *= *E*[*V*/*R*|*R *> 0], the expectation conditional on at least one rejection, appears in [2]. Other forms of *FDR *have been proposed such as the conditional *FDR *[2], *cFDR*, defined as the expected proportion of false positives conditional on the event that *R *= *r *rejected have been observed : *cFDR*(*r*) = *E*[*V*|*R *= *r*]/*r*. This would answer to the question "What proportion of false positives may I expect in my top list of *r *genes?". Under independence and identical distribution in a Bayesian setting it is proved in [23], that *pFDR*, *cFDR *and the marginal *FDR*, *mFDR *= *E*[*V*]/*E*[*R*] [2], all coincide with *p*_0_*α*/*F*(*α*) at the cutpoint *α*, cf. (6).

Instead of *p*-values it has been suggested in to calculate *q*-values that verbally have the following meaning for an individual gene [2,9]:

*The q-value for a particular gene is the minimum false discovery rate that can be attained when calling all genes up through that one significant *[9].

These *q*-values can be used to determine a cut-off similar to the classic 5% cut-off for univariate tests developed in statistics long ago. But in many applications one should not be too rigid about any particular value, since the emphasis often is on discovery rather than hypothesis testing: we generate hypotheses worthy of further investigation. Thus the balance between false positives and false negatives will be crucial: Rather than keeping the risk of erroneously selecting *one *individual gene at a fixed level, it is the decision involving *thousands *of genes given the amount of genes we can follow up on that is the focus, and where *both *types of error must be considered. The *q*-value does not fully address this problem, but nevertheless represents an improvement over the classical multiple test procedures in these applications.

More mathematically the *q*-value can be expressed as



Taking minimum in (7) enforces monotonicity in *p*_*i*_, so that the *q*-value will be increasing (non-decreasing) in the observed *p*-value. If the FDR is non-increasing, as it should, then *q *(*p*_*i*_) = *FDR *(*p*_*i*_).

Additionally, the *FDR *offers a framework for power and sample size calculations, see [24] and the new developments in [25].

## Results

Eight estimates of *p*_0 _and six of FDR (based on six of the former) were tested on both simulated data and real data. The differing numbers are motivated below.

Next follows a list of six *p*_0 _estimation methods and the corresponding R functions. The six were

1. the beta-uniform model (BUM) [10], which fits a mixture of a uniform and a beta distribution to the observed *p*-values; function *ext.pi*.

2. spacing LOESS histogram (SPLOSH) [11], which fits a non-parametric spline that estimates the logarithm of the pdf;function *splosh*.

3. the Lowest SLope estimator (LSL) [12,26] ;function *fdr.estimate.eta0*.

4. the smoother [9], which fits a spline to a function of a cut-off value, namely the proportion of *p*-values greater than that cut-off divided by the expected proportion under the uniform distribution;function *qvalue*.

5. the bootstrap least squares estimate (bootstrap LSE) [3], which is related to the previous estimate;function *qvalue *or *estimatep0*.

6. the Successive Elimination Procedure (SEP) [13];selects a subset which represents the null distribution by behaving like a uniform;function *twilight*.

7. a new method based on a moment generating function approach (*mgf*);function *p0.mom*.

8. a Poisson regression approach (PRE); an adaptation of [27,28];function *p0.mom*.

The bootstrap estimate and *mgf *did not participate in the calculation of FDR. The smoother gives the basically same value as the bootstrap estimate, and mgf is unnecessarily conservative for lower values of *p*_0_, compared to PRE.

The six were

1. BUM FDR (based on BUM;function *bum.FDR*)

2. BH FDR (based on LSL and function *fdr.control*).

3. SPLOSH FDR (based SPLOSH;function *splosh*)

4. smoother FDR or R function *qvalue *[9] (based on the smoother)

5. SEP fdr (based on SEP;function *twilight*)

6. the new method pava FDR (based on PRE;function *pava.fdr*)

For brevity mgf, PRE and pava FDR will all be referred to as new methods. It would be more exhaustive to say that PRE is a minor modification of an existing method [25] applied to *p*-values rather than test statistics and provided as a new implementation in R; and that pava fdr is based on [29] with local splines replaced by isotonic regression and provided as a new R function. On the other hand *mgf *seems quite new. More details follow in **Methods**.

For reference some graphs include an estimate of the SEP local FDR, defined as *LFDR*(*p*) = *p*_0_/*f*(*p*), estimating the probability that a gene whose *p*-value equals *p *is a false positive. Furthermore, the ouput from R function *locfdr *applied to the real life data (with nulltype = 0, i.e. a standard normal distribution which has cdf Φ, see **Methods**) and the transformed *t*-test statistics : *Z *= Φ^-1^(*F*(*t*)), and *F *the *t*-test distribution (details below) gives perspective on the other methods and opens up an alternative route to making inferences. This function produces an estimate of the local FDR as a function of the transformed test statistic *Z *[25]. In that same reference the author argues in favour of the cutpoint LFDR ≤ 0.2, which implies quite high posterior odds in favour of the non-null case : (1-*p*_0_)*f*_1_/*p*_0_*f*_0 _≥ 4.

### Simulated data

Two simulation models were used: one generating values independent between genes and the other generating observations displaying clumpy dependence [14,30].

Simulated independent data corresponding to two groups of four samples each were generated 10,000 genes 400 times for each of the true *p*_0 _values ranging from 0.5 to 0.99 using the R script from [5]. Normal distributions were chosen randomly from the ones in Table 1. The cases of DEGs correspond to the power of 41%, 54% and 97% given a *t*-test with a significance level of 5% [12]. Briefly, the script generated a mixture of normal distributions and for each run calculated *t*-tests to obtain *p*-values.

**Table 1:** Simulation of independent data. Denote by N(*μ*, *σ*) a normal distribution with mean *μ *and standard deviation *σ*. For each DEG one of the above three scenarios was chosen with equal probabilities. For the rest both groups follow the same distribution chosen randomly from the 'Group 1' column. The scenarios are such that the power to detect the regulation with a 5% two-sided *t*-test ranges from small to large given two groups of size four.

Scenario	Group 1	Group 2	Power
1	N(6, 0.1)	N(6.1, 0.1)	0.19
2	N(8, 0.2)	N(8.5, 0.2)	0.79
3	N(10, 0.4)	N(11, 0.7)	0.47

To generate dependent data the protocol from [14] was followed. This generates data following clumpy dependence in the sense of [30] such that blocks of genes have dependent expression. First a logarithmic normal distribution with mean 1.5 and standard deviation 0.3 generated a profile for each gene. Denoting by *N*(*μ*, *σ*) a normal distribution with mean *μ *and standard deviation *σ*, random errors following a standard normal distribution *N*(0,1) were added. To create dependencies genes were partitioned into sets of 50 and for each sample the same term from a *N*(0, 1) distribution was added to the expression of each gene in the set. Finally, genes were randomly assigned to become DEGs with probability 1-*p*_0 _and for each gene a regulation term following either *N*(0.5, 0.2) or *N*(0.7, 0.2), with equal probabilities, was added to the expression of one of the groups of samples of size 30. The power to detect either of these two alternatives with a *t*-test at the 5% significance level equals 31% and 50%, respectively. The procedure generated for each of 400 iterations a set of observations of 10,000 genes. The protocol gives rise to high correlation within the blocks of 50 (on average on the order of 0.5). Results for smaller or weakly dependent datasets appear in the Additional file. Here weakly dependent means that the clumpy dependence term follows a N(0,1/20) distribution (correlation within blocks slumps to 0.003 on an average).

**Figure 1:**
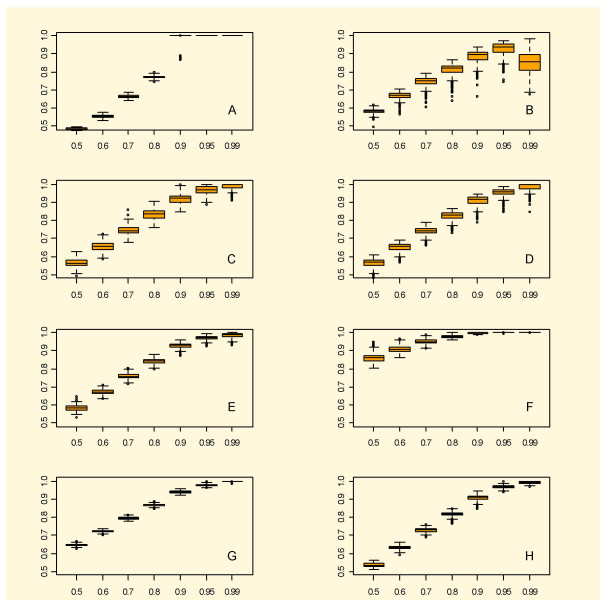
Boxplots of the results from eight methods for estimating p_0 _using simulated independent data. A simulation model of real-life microarray data was used to give data where the expected proportion of unchanged genes was set at 50, 60, 70, 80, 90, 95 or 99%. The central box of the boxplot represents the data between the 25% and 75% percentiles with the median represented by a line. Whiskers go out to the extremes of the data, and very extreme data appear as points. The abscissa shows the expected proportion unchanged and ordinata the estimate. Legends : A – BUM;B – SPLOSH; C – smoother; D – bootstrap; E – SEP; F – LSL; G – mgf; H – PRE. A and B underestimate in the low range and in the high range, respectively; C and D appear very similar with a sound overestimation and some high variance; E appears stable and reliable; F gives a stable and quite conservative estimate; G overestimates and varies very little; G is stable and gives a small degree of overestimation.

With the simulated independent data all methods for estimating *p*_0 _perform rather well, see Table 2 and 3, and Figure 1.

**Table 2:** Over-all results of simulations of independent data. Data sets with *p*_0 _ranging from 0.6 to 0.99 were simulated. The summary statistics of the absolute difference between target value and its estimate show a rather varying performance for all methods, with PRE having the smallest bias and variation.

	BUM	SPLOSH	*smoother*	*bootstrap*	*SEP*	*LSL*	*mgf*	PRE
*Mean*	0.039	0.061	0.038	0.036	0.045	0.18	0.072	0.022
*Sd*	0.048	0.078	0.032	0.034	0.032	0.12	0.048	0.016

**Table 3:** Detailed statistics on the estimates of *p*_0 _based on simulations of independent data. The displays the mean bias (true – estimated) and standard deviation of estimates for each level of true *p*_0_.

***True p*_0_**		0.5	0.6	0.7	0.8	0.9	0.95	0.99
***BUM***	mean bias	0.013	0.043	0.038	0.028	-0.090	-0.050	-0.010
	Sd	0.0044	0.0074	0.0072	0.0076	0.033	0.0021	0.0010

***SPLOSH***	mean bias	-0.083	-0.068	-0.043	-0.012	0.018	0.026	0.14
	Sd	0.014	0.020	0.028	0.034	0.040	0.041	0.063

***QVALUE***	mean bias	-0.066	-0.057	-0.046	-0.034	-0.019	-0.015	0.0012
	Sd	0.022	0.022	0.023	0.027	0.027	0.024	0.0160

***Bootstrap LSE***	mean bias	-0.065	-0.054	-0.040	-0.025	-0.0067	-0.0037	0.0057
	Sd	0.023	0.023	0.021	0.024	0.026	0.024	0.023

***SEP***	mean bias	-0.084	-0.072	-0.059	-0.043	-0.028	-0.020	0.0068
	Sd	0.014	0.013	0.014	0.013	0.013	0.012	0.014

***LSL***	Mean bias	-0.36	-0.31	-0.25	-0.18	-0.096	-0.049	-0.010
	Sd	0.025	0.019	0.014	0.0086	0.0036	0.0022	0.0010

***mgf***	mean bias	-0.15	-0.12	-0.095	-0.067	-0.040	-0.027	-0.0095
	Sd	0.0052	0.0052	0.0051	0.0056	0.0056	0.01098	0.0018

***PRE***	mean bias	-0.036	-0.033	-0.028	-0.018	-0.0078	-0.018	-0.0018
	Sd	0.0098	0.010	0.0097	0.012	0.016	0.0080	0.0067

The new methods *mgf *and PRE were very competitive on these data, and had both low bias and variation, excluding *mgf *at the 0.5 and 0.6 level. Since *mgf *tends to overestimate *p*_0 _rather much in the lower range, one may prefer PRE. For practical purposes though overestimation is desirable and enables control of the error rate (exact control in the terminology from [31]).

The smoother and the bootstrap had good and quite similar performance. They give more or less the same variation and bias over the whole range. This variation can be a bit high though, especially when comparing to PRE.

In the higher range BUM gives a crude estimate of the true *p*_0_. In a certain lower range however it underestimates. As we can see in Figure 1, however, the method considerably overestimates *p*_0 _in the higher range, which brings down the power to detect DEGs.

SPLOSH has the advantage of fitting the observed distribution quite well, judging from some tests (data not shown), compare also [11]. This enables a Bayesian analysis as in (3). However, it does the fitting of the *p*-values close to 0 sometimes at the expense of the accuracy concerning the values at the other end, thus misses the plateau and the minimisation in (10) will give a misleading result. In particular, this tends to happen when there are few DEGs. As we can see in Figure 1 the method underestimates *p*_0 _at the higher range (*p*_0 _≥ 0.9), which is worrisome and may lead to underestimation of the error rate, which is undesirable for a method of statistical inference.

From Tables 2 and 3 we can see that PRE has the best over-all performance, followed by the smoother and the bootstrap. This does not however imply that the other methods could not be considered. The results vary quite a lot depending on the value of *p*_0_: LSL is quite competitive for *p*_0 _= 0.99, but too conservative for *p*_0 _= 0.5.

With simulated data it is possible to calculate the actual false discovery rate. Figure 3 shows boxplots based on the simulated independent data. Here *qvalue *and pava FDR stand out as most reliable. Especially BUM FDR has severe problems, often over the whole range of cut-offs, while SPLOSH FDR does rather well.

**Figure 2:**
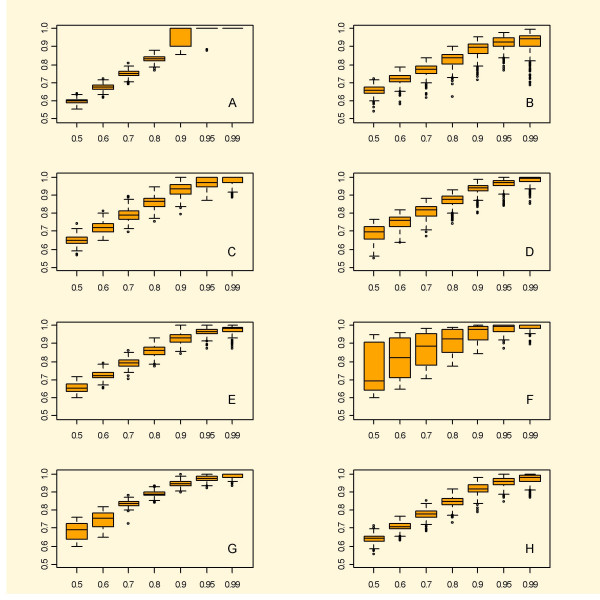
Boxplots of the results from eight methods for estimating *p*_0 _using simulated dependent data. Legend as in Figure 1. There is a general trend towards greater overestimation compared to the independent case.

The dependent data gave a slightly different picture. Most importantly, the variation increased considerably. The results concerning *p*_0 _appear in Figure 2. Interestingly underestimation becomes less of a problem for SPLOSH this time. Relatively speaking BUM did better this time, and BH comes out worst. As far as estimation of *p*_0 _goes BUM does quite well, see Tables 4 and 5. The method manages to overestimate at high *p*_0 _by consistently outputting 1. At *p*_0 _= 0.95 the estimate was nearly always equal to 1. Both qvalue and bootstrap underestimated by a small amount at *p*_0 _= 0.99. SEP emerges as a sharp contender, but suffers from a small underestimation at *p*_0 _= 0.99. In fact, all methods except BUM give underestimation at *p*_0 _= 0.99, and for SPLOSH it amounts to almost 7%.

**Table 4:** Over-all results of simulations of dependent data. Data sets with *p*_0 _ranging from 0.6 to 0.99 were simulated. The summary statistics of the absolute difference between target value and its estimate show a rather varying performance for all methods, with BUM now having the smallest bias and variation with PRE in second place.

	BUM	SPLOSH	*Smoother*	*Bootstrap*	*SEP*	*LSL*	*Mgf*	PRE
*Mean*	0.054	0.075	0.07.3	0.085	0.071	0.125	0.091	0.064
*Sd*	0.035	0.086	0.062	0.076	0.062	0.124	0.069	0.060

**Table 5:** Detailed statistics on the estimates of *p*_0_**based on simulations of dependent data. The table displays the mean bias (true – estimated) and standard deviation of estimates for each level of true *p***_0_.

***True p*_0_**		0.5	0.6	0.7	0.8	0.9	0.95	0.99
***BUM***	mean bias	-0.09885	-0.0744	-0.0524	-0.0308	-0.0525	-0.0493	-0.0100
	Sd	0.01555	0.0167	0.0169	0.0194	0.0520	0.0088	0.0010

***SPLOSH***	mean bias	-0.15811	-0.1179	-0.0710	-0.02570	0.0180	0.0318	0.06761
	Sd	0.02702	0.0309	0.0338	0.0398	0.0423	0.0379	0.0560

***QVALUE***	mean bias	-0.15089	-0.1217	-0.0895	-0.0593	-0.0307	-0.0142	0.0093
	Sd	0.02661	0.0306	0.0302	0.0345	0.0368	0.0309	0.0255

***Bootstrap LSE***	mean bias	-0.18009	-0.1499	-0.1080	-0.0691	-0.0313	-0.0126	0.0084
	Sd	0.04242	0.0382	0.0351	0.0326	0.0299	0.0269	0.0232

***SEP***	mean bias	-0.15525	-0.1238	-0.0915	-0.0584	-0.0277	-0.0134	0.0138
	Sd	0.02419	0.0239	0.0241	0.0265	0.0269	0.0203	0.0201

***LSL***	Mean Bias	-0.2710	-0.2203	-0.1667	-0.1110	-0.0554	-0.0292	0.0017
	Sd	0.13333	0.1119	0.0902	0.0671	0.0410	0.0221	0.0165

***Mgf***	mean bias	-0.18322	-0.1468	-0.1349	-0.0898	-0.0460	-0.0235	0.0007
	Sd	0.04633	0.0401	0.0140	0.0153	0.0160	0.0153	0.0128

***PRE***	mean bias	0.14088	-0.1091	-0.0770	-0.0442	-0.0152	-0.0021	0.0194
	Sd	0.02300	0.0239	0.0239	0.0291	0.0310	0.0269	0.0248

**Figure 4:**
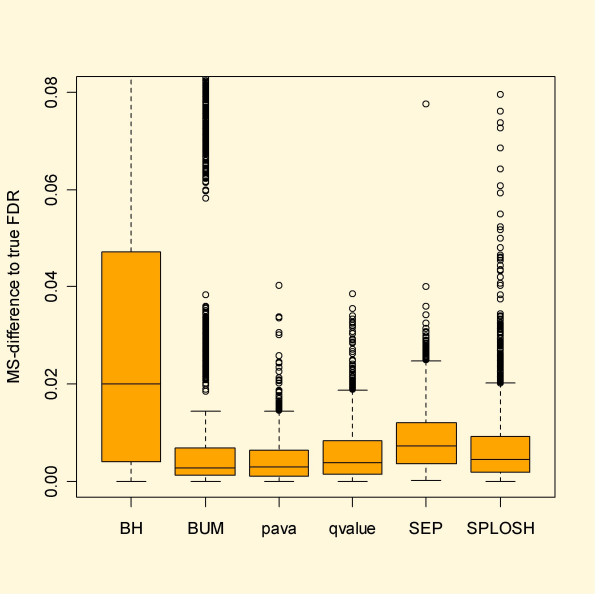
Simulations of FDR based on dependent data. All methods perform worse on dependent data. All except BH perform reasonably well. In the case of BH the problem lies mainly in the lower range of *p*_0_.

The results concerning FDR appear in Figure 4. This time the BUM method captures FDR in a competitive way, with the lowest median error but at the same time the second worst mean error. Again the results for pava FDR appear quite stable and accurate, giving the second lowest median error and the lowest mean error.

Results for 300, 5,000 and 10,000 simulated weakly dependent genes appear in the Additional file. Briefly, they resemble those of the independent case. For 300 genes however the variation is such that the value of using these methods seems doubtful.

### Real data

#### Data from Golub et al

**Figure 5:**
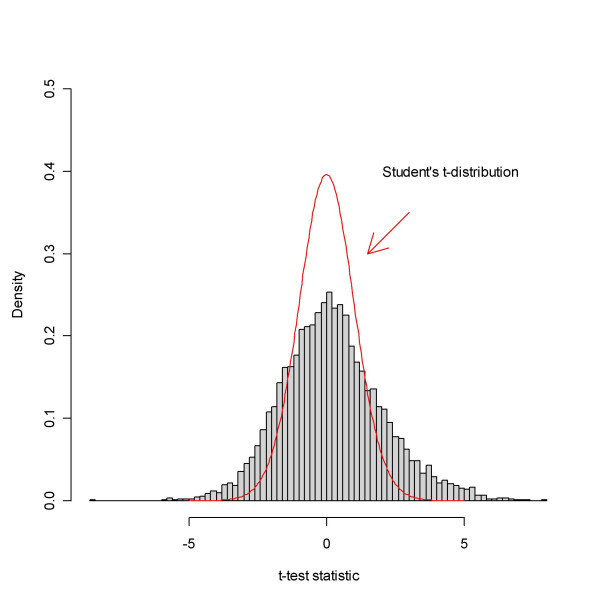
The empirical distribution of the *t*-test statistic based on the data from Golub *et al. *The test compares the sample types AML and ALL. A comparison with the theoretical null distribution, Student's *t*-distribution, hints that there are too many extreme values to be accounted for by pure chance. In fact, evidence is that a substantial part of the genes differ.

These data represent a case where there are many DEGs. The data set concerning two types of leukaemia, ALL and AML, appeared in [32,33]. Samples of both types were hybridised to 38 Affymetrix HG6800 arrays, representing 27 ALL and 11 AML. In reference [32] 50 genes were identified as DEGs using statistical analysis. The data consisted of Average Difference values and Absolute Calls, giving for each gene (probe set), respectively, the abundance and a categorical assessment of whether the gene was deemed Absent, Marginal or Present. The Average Difference values were pre-processed as in [5], and the proportion of samples where each probe set was scored Present was calculated, giving a present rate. A permutation *t*-test was used to compare ALL and AML. Figure 5 shows a histogram of *t*-test statistics revealing a bell-shaped region around the origin and large tails, indicating that a substantial part of the genes are DEGs.

**Figure 6:**
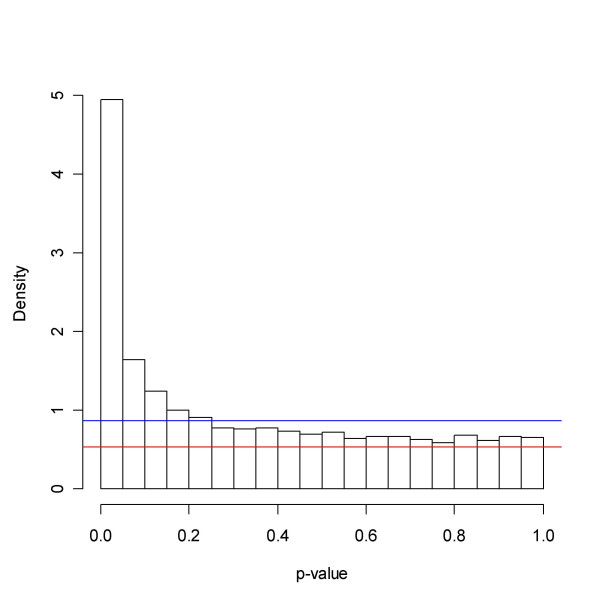
Golub *et al. *data: *p*-values. The blue and red lines represent the minimum and maximum estimate of *p*_0 _obtained from the six methods under investigation. The plateau to the right resembles a uniform distribution, and most likely to a large degree represents a population of unchanged genes.

**Figure 7:**
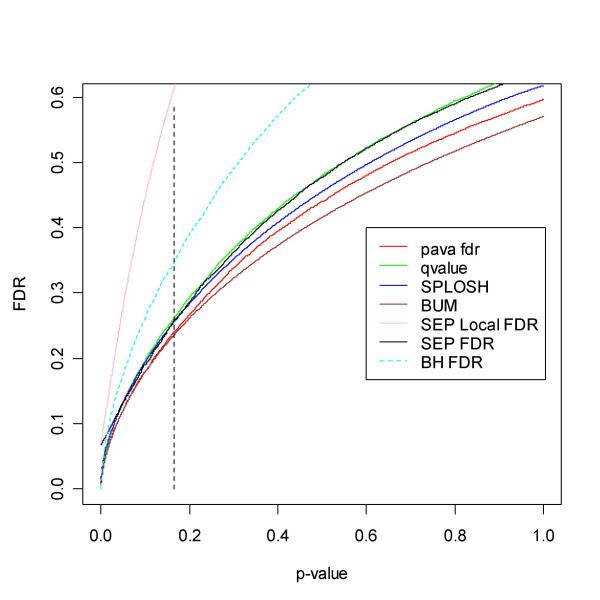
FDR for the Golub *et al. *[32] data. The dotted line represents a cut-off that minimises the number of false positives and false negatives, summing (4) and (5) using the smoother estimate. It would make sense to consider choosing a threshold in this region. The line corresponds to the 2920^th ^ordered *p*-value and a q-value of just above 0.2. All methods agree rather well. For reference the local FDR of [29] has been added. As expected it exceeds all others, and is caught up at zero by its FDR counterpart SEP FDR. However, they meet at a level well above other methods.

**Figure 8:**
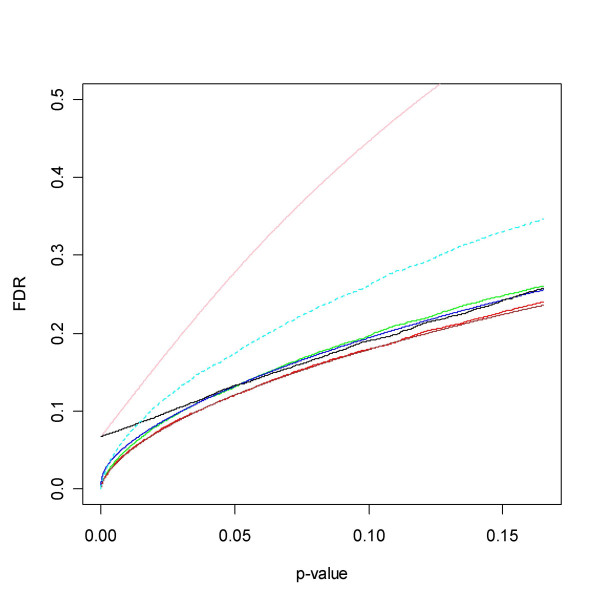
A blow-up of the region to the left of the dotted line in Figure 7. The SEP estimates of FDR and LFDR join at roughly 0.07.

Visual inspection of Figure 6 showing the *p*-value distribution also suggests that many genes are altered. The methods tested vindicate this : BUM, SPLOSH, bootstrap, *qvalue*, mgf, PRE, SEP, LSL give the estimates 0.57, 0.62, 0.65, 0.65, 0.69, 0.60, 0.64 and 0.86, respectively. Thus, roughly 35% of the genes are regarded as changed. The function *qvalue *finds 873 probe sets with a q-value less than 5%. The estimated of FDR appear in Figures 7 and 8, and show a great deal of concordance between methods. The curves rise to the respective estimate of *p*_0 _and and in doing so slowly diverge. SEP approaches roughly 0.07 in a vicinity of zero, while the other methods continue the decline.

**Figure 9:**
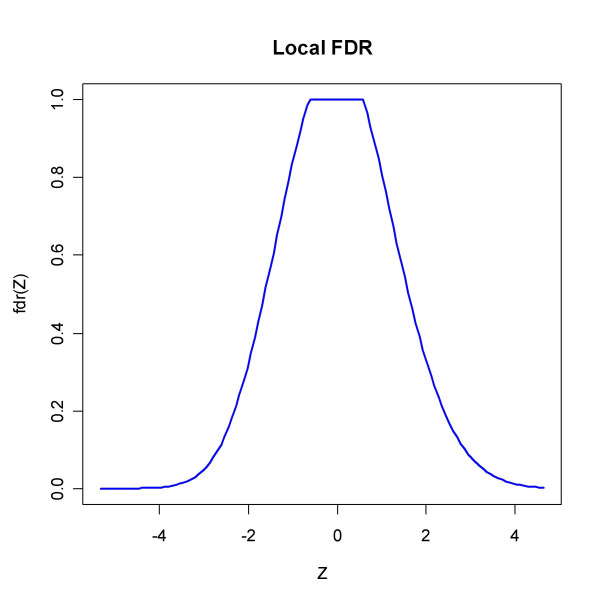
The local fdr from *locfdr *applied to data from [32]. Using Z statistics based on the transformed test statistics (in R code Z = qnorm(pt(tt, df = 36)), pct0 = 0.5 and a standard normal density as null type), the function outputs the estimate *p*_0 _= 0.66 and a plot of the local fdr which reflects the skewness of the test statistic distribution. The requirement that LFDR be less than 0.2 would identify 1198 genes as significant.

The function *locfdr *applied to *Z *= Φ^-1 ^(*F*_36_(*t*)) with *F*_36 _the cdf of the *t*-distribution with 36 degrees of freedom gives the estimate of *p*_0 _0.66 and outputs Figure 9 which bears witness of the skewness and of the different local false discovery rates in the two tails of the distribution.

Removing probe sets with less than 20% present rate will leave us with 2999 probe sets, and *qvalue *indicates that there are 977 of them that are significant with a q-value less than 5% (*p*_0 _= 0.47). In general it is wise to remove probe sets with low presence rate prior to analysis, since this will make the inference more reliable, compare [20]. Doing so will most likely produce more true positives.

#### Data from Spira et al

Next let us turn to a case where there are rather few DEGs. In [34] the results from a microarray experiment where bronchial epithelial brush biopsies have been hybridised to Affymetrix U133A arrays are presented. The biopsies come from three different subject categories: Current smokers, Never smokers and Former smokers. The cel intensity files were downloaded from the NCBI Gene Expression Omnibus (accession no. GSE994) [35]. The Bioconductor package *affy *[19] was used to normalise intensities with the quantile method, and to calculate the RMA measure of abundance. The function *mas5calls *in *affy *output absolute calls.

Here we will take a brief look at the comparison between Former smokers and Never smokers. The comparison may help identify genes that remain changed after smoke cessation. A fuller analysis would include more analyses, such as the Current smokers vs. Former smokers comparison, and possibly also adjust for the fact that Former smokers tend to be older than Never smokers (Mean Age 45 and 53 years, respectively).

**Figure 10:**
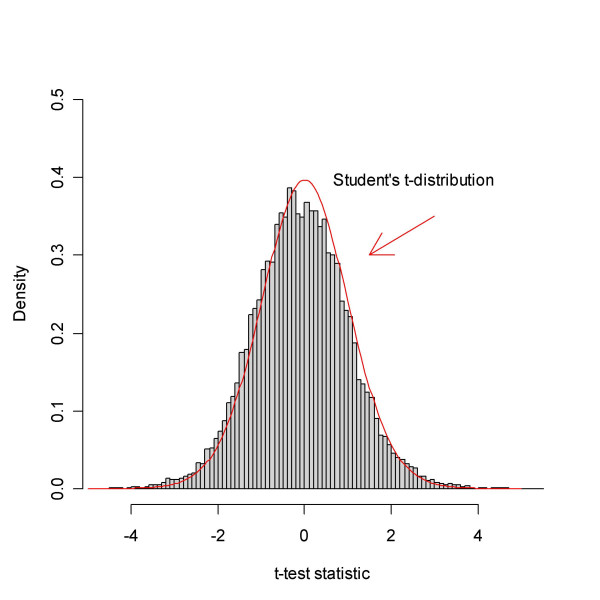
Spira et al. data: Former smokers vs. Never smokers *t*-test statistic. The shape of the histogram indicates that there are more genes that are more highly expressed in Former smokers than the other way round, but on the whole there are rather few DEGs. Indeed, the estimates of *p*_0 _vindicate this and suggest that less than 10% of the genes are DEGs. Compare Figure 11.

Graphical representations of the results appear in Figures 10 through 14.

**Figure 14:**
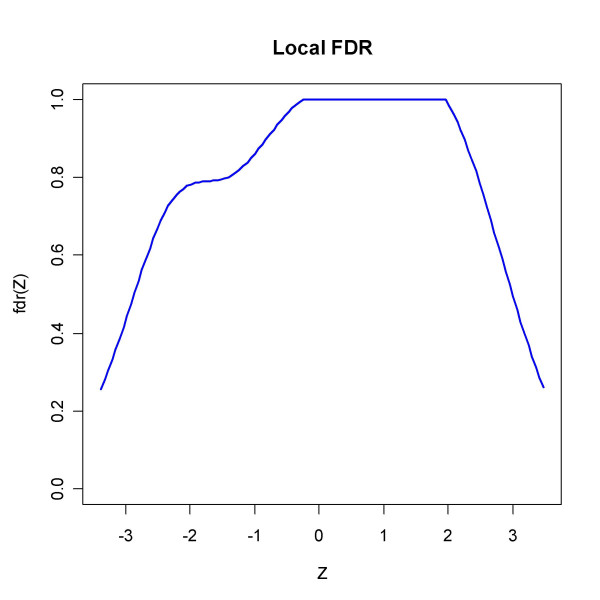
Output from *locfdr *applied to the Spira *et al. *data. An estimated proportion of p_0 _= 0.96 were not changed. The input variable was a transformed *t*-test statistic Z (in R code Z = qnorm(pt(tt, df = 39))).

**Figure 11:**
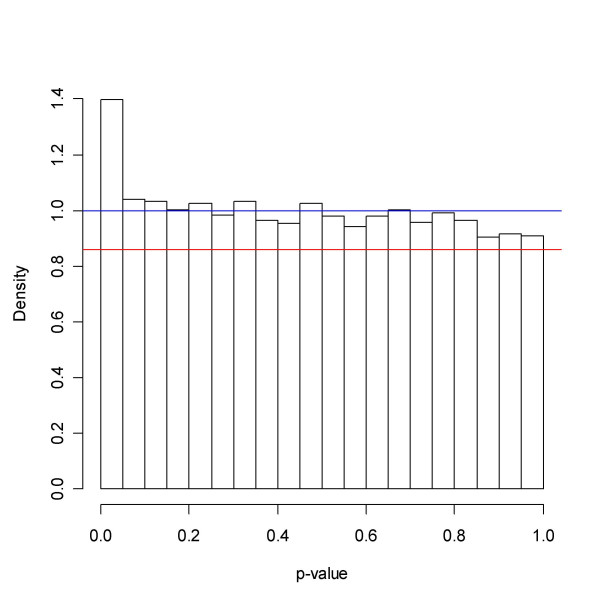
Spira *et al *data: Former smokers vs. Never smokers *p*-values. The binned densities in the histogram coupled with the inequality (9) points to a value of *p*_0 _of more than 0.9. The red and blue lines represent the maximum and the minimum estimate obtained from the methods under investigation.

**Figure 12:**
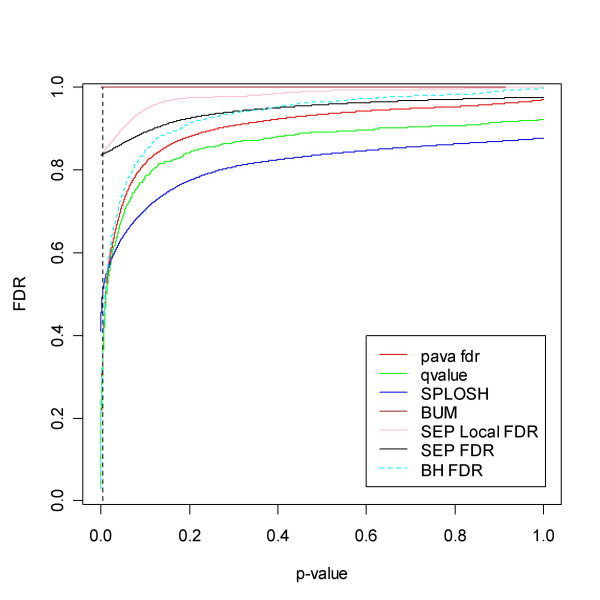
FDR calculated from the Spira *et al. *data. The dotted line indicates where the optimal cut-off is estimated to be in the sense of minimising the sum of false positives and false negatives. At this point the FDR of BH, pava and qvalue is close to 0.4, and corresponds to the 298^th ^smallest *p*-value. This high proportion of false positives may be acceptable given some high throughput validation procedures, but may be unacceptable in other contexts. Note that BUM produces the overly pessimistic estimate FDR ≡ 1, and hence appear at the upper limit of the graph. For comparison the local FDR [13] has been added to the graph.

**Figure 13:**
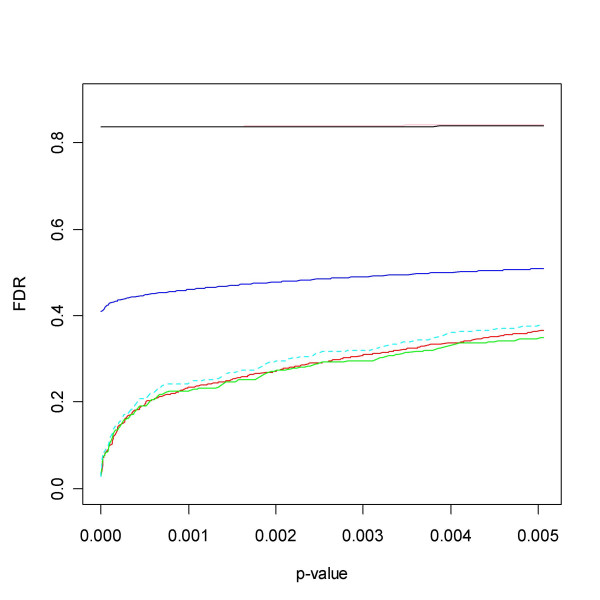
Blow-up of region to the left of dotted line in Figure 12. Legend as in Figure 11A. SEP Local FDR and SEP FDR behave similarly in a vicinity of zero. These two methods indicate that the relative frequency of false positives stabilises to a value above 80% when the cut-off approaches 0. SPLOSH, on the other hand, approaches 0.4. Note that BUM does not appear in the graph.

Figure 10 suggests that rather few genes are changed. The methods give the following estimates of *p*_0 _: bootstrap 0.92, mgf 0.97, PRE 0.97, LSL 1.00, SEP 0.98, SPLOSH 0.88, BUM 1.00 and smoother 0.92, compare Figure 11. The estimates of FDR appear in Figures 12 and 13, and show some separation between methods. The trio BH, pava and qvalue largely agree. SEP LFDR and FDR stabilise at a value above 0.8 when the cut-off approaches zero. This is consistent: If the local FDR levels off, then the Averaging Theorem, see **Methods**, implies that so will the FDR. However, this level may seem incompatible with the assessment of SEP that 2% of the genes are truly changed. A rough calculation, replacing f by a histogram, would yield the estimate of LFDR(p) = p_0_/f(p) = 0.22 in a vicinity of p = 0.00025. Neither does it agree with *locfdr*, see Figure 14. Most likely the spline function employed by SEP is playing tricks here. SPLOSH approaches 0.4 in a vicinity of zero. BUM returns the estimate FDR ≡ 1, which is consistent with its estimate of *p*_0_, but does not seem to agree with Figure 11. The method may display numerical problems with these data. The function *qvalue *finds one gene significant with a *q*-value less than 5%. However, if we restrict attention to the probe sets with at least 20% present rate (9841 genes) there are fifteen genes fulfilling the criterion.

Figure 14 displays the graphical output from *locfdr*, where the LFDR seems to approach zero in the tails, contradicting SEP, SPLOSH and BUM, but essentially agreeing with the concordant trio BH, pava and qvalue.

## Discussion

Over-all results will lump together performance under different conditions and may thus be less relevant for a particular application. For instance, in practice the performance for high *p*_0 _will probably matter more than that for lower values. When many genes are changed the cutpoint will likely be chosen based on other criteria than FDR, and hence the difference between methods becomes less relevant. However, the detailed results presented here should give the practitioner some guidance as to what methods could be considered. Looking at the *p*-value histogram one can find some decision support in the choice of method. Comparing the output from several methods provides further clues.

All the methods performed worse on the dependent data; both the estimate of *p*_0 _and FDR suffered. To some extent that may be due to the lower mean power of the alternatives in that simulation model. However, the methods were derived under the assumption of independence and the small difference in mean power of 0.08 does not explain the great deterioration in most methods. Indeed, for simulated datasets with weak dependencies the results came close to the independent case, see the Additional file.

Through all tests PRE and pava FDR proved quite successful. Of the methods for estimating FDR, *qvalue *has the advantage of a well-documented and good track record, and behaves well here. BUM displays a varying performance, but does handle dependent data well. In practice it will be difficult to know where on the scale from independence to strong clumpy dependence a particular dataset will rate, if indeed it follows clumpy dependence at all. LSL, and BH, have some problems, but on the other hand they arise mainly at low *p*_0_, where they probably matter the least. As noted above regarding the Spira et al. data, SEP LFDR and FDR stabilised at a value above 0.8 when the cut-off approaches zero. In other tests SEP performed well, particularly with independent data. BUM in this case produced the estimate FDR ≡ 1 which can hardly reflect the truth.

The *locfdr *method offers the possibility to choose between three different null type distributions. The choice of the null type N(0,1) produced *p*_0 _estimates similar to those of the other methods. The need to specify the transform *m *may seem like an obstacle. But in many situations a parametric test statistic with a known null distribution exists. Alternatively, *m *could be identified by modelling a bootstrap distribution [25].

All the described methods assume the *p*-values were obtained in a reliable fashion, e.g. by a warranted normal approximation, a bootstrap or a permutation method. Reference [10] describes a case when a two-way ANOVA F-distribution was used when the distributional assumptions were not met. The estimate of *p*_0 _gave an unrealistic answer. When permutation *p*-values were used instead their method gave a more realistic result. One always has to bear this caveat in mind. To further complicate matters, the permutation of sample labels approach is no panacea if the independence between samples assumption does not hold true, as detailed in [27]. (Let us follow the usual convention that genes come in rows of the data matrix, and samples in columns.) Permuting within columns provided some remedy there. Misspecifying the null distribution will jeopardize any simultaneous inference, whether based on FDR or not. It may pay off to consider the correlation structure in data, both in view of this finding and in view of the different performance of methods depending on the strength of correlations.

The q-value *q*(*p*_*i*_) has been criticised for being too optimistic in that it weighs in also genes that are more extreme than *i *when calculating the measure. Note that a similar criticism could be levied against the classical *p*-value: the *p*-value gives the probability under the null hypothesis of observing a test statistic at least as extreme as the one observed. Also, there is no clear stable, reliable and tested alternative. This is not to say that the q-value is unproblematic, but it still has been studied and used much more than e.g. the local FDR, which may suffer from high random variation, see examples in [29]. Other examples from **Results **section give evidence of stability. Contrary to what one may anticipate the FDR is not always more stable than LFDR [25]. The concept of a local FDR seems quite interesting and may lead the way towards improved inference, and it begs a thorough investigation of the various recently published options.

To avoid pit-falls in the inference one must use the total information obtained from *p*_0 _and the FDR or q-value curve, see also Storey in the discussion of [31]. There is not one cut-off in terms of q-value that will suit all problems. Take the case of Figure 12, where one will have to accept a high FDR in order to find any DEGs. At the other end of the spectre, in Figure 7, the cut-off can be much more restrictive. The choice of cut-off must be made with a view to one's belief regarding *p*_0_, and calculating the sum of (4) and (5) to assess to total of false positives and false negatives gives further guidance in this choice. In general it makes sense to choose a cut-off in the region [0, *α*_*min*_], where *α*_*min *_is the value which minimises the total relative frequency of errors committed *FP*(*α*)+*FN*(*α*), see (4) and (5). However, since false positives and false negatives have different consequences with possibly different losses, it is difficult to state an algorithm that would cover all scenarios.

## Conclusion

This article deals in the main with a simple frequentist framework for the analysis of microarray experiments. The conclusion is that the concept of the proportion of unchanged genes and the related concept of a q-value or false discovery rate are practical for such analysis. Furthermore, there exists open source code that implements methods that address the needs of the practitioner in this field. New methods gave evidence of improved performance, allowing better control of the error rate and thus enabling a more careful identification of DEGs. Issues still remain and improvements will probably appear over the next couple of years, but as a provisional solution these methods have much to offer.

## Methods

The current article focuses on the two-component model. Other points of view exist. In reference [25] the two-component model is reshaped into a conceptually attractive one-group model allowing a continuum of effects.

Denote the pdf of *p*-values by *f*, the proportion of unchanged by *p*_0 _and the distribution of the *p*-values for the changed genes by *f*_1_. Then the pdf of *p*-values may be written as

*f*(*x*) = *p*_0 _× 1 + (1 - *p*_0_)*f*_1_(*x*)     (8)

using the fact that *p*-values for the unchanged genes follow a uniform distribution over the interval [0,1]. This model is unidentifiable without further assumptions, e.g. that p-values in a vicinity of 1 only represent unchanged genes. From the non-negativity of pdf's, clearly

*f*(*x*) ≥ *p*_0 _    (9)

This leads to the estimate based on the minimum of the estimated pdf [1]



see also Figure 6. In most cases the minimum in (10) will occur for some *x *close to or at 1. Hence (10) will in these cases agree well with an estimate of *f*(1). If one has reason to believe that *p*_0 _is close to 1, it may pay off to replace (10) by the 25% percentile or simply put the estimate equal to 1, in order to make overestimation more likely.

### LSL

Let *R*(*α*) = # {*i *: *p*_*i *_≤ *α*}, the number of rejected given the cut-off *α*. In [36] the approximation

*N *- *R*(*α*) ≈ *E*[*N *- *R*(*α*)] ≈ *N*_0_(1 - *α*)

for small α and *N*_0 _= *Np*_0 _the number of true null hypotheses appears. Consequently, (*N *- *R*(*p*_(*i*)_)/(1 - *p*_(*i*)_) = (*N *- *i*)/(1 - *p*_(*i*)_) will approximate *N*_0_, which lead the pioneering authors to consider plotting 1 - *p*_(*i*) _against *N *- *i*, thus giving them an estimate of *N*_0_. In [26] the Lowest SLope estimator (LSL) of *N*_0 _based on the slopes *S*_*i *_= (1 - *p*_(*i*)_)/(*N *- *i *+ 1) is presented. Starting from *i *= 1, the procedure stops at the first occurrence of *Si*_0 _<*Si*_0_-1, and outputs the estimate



In [12] the two above estimates are presented, derived and compared together with a method called Mean of Differences Method (MD). MD and LSL are motivated by assuming independence and approximating the gaps *d*_(*i*) _= *p*_(*i*) _- *p*_(*i*-1) _(define *p*_(0) _= 0 and *p*_(*N*+1)) _≡ 1) with a *Beta*(1, *N*_0_) distribution, which has expectation 1/(*N*_0 _+ 1). This expectation may be estimated by the inverse of a mean of the form



MD proceeds downward and chooses *i*_0 _equal to the first *j *satisfying



Of these three methods LSL and MD give very similar results, and outperform their predecessor [12].

LSL is available as function *fdr.estimate.eta0 *in package GeneTS [18] with the option method= "adaptive".

### The smoother

**Figure 15:**
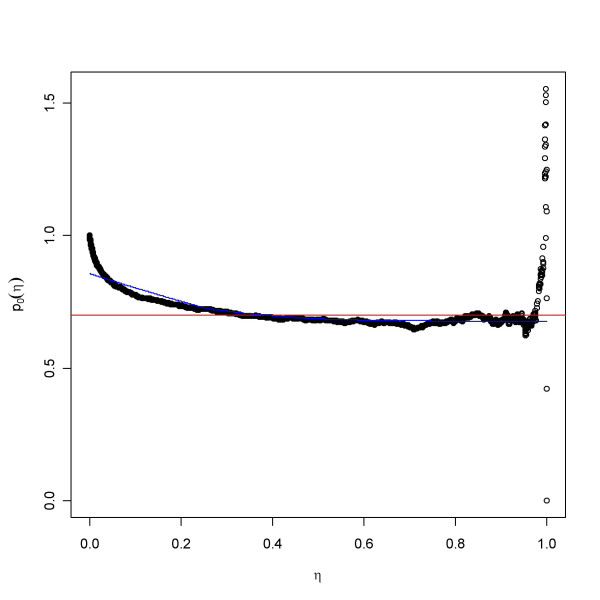
The smoother estimate of *p*_0_. This is based on the ratio *p*_0_(*η*) = (1 - *F*_*e*_(*η*))/(1 - *η*), the observed ratio proportion of *p*-values greater than *η *to the proportion expected from the uniform distribution. The data were simulated with *p*_0 _= 0.6. The red line represents the true value, while the blue gives a smooth curve representation of *p*_0_(*η*) approaching a limit close to the true value.



A method here referred to as the smoother appeared in [9]. This method, like all presented, is based on a comparison of the empirical *p*-value distribution to that of the uniform distribution. There will likely be fewer *p*-values close to 1 in the empirical than in the null distribution, which is a uniform. The ratio of the proportion of *p*-values greater than some *η *to the expected proportion under the uniform distribution, 1-*η*, will give a measure of the thinning of observed *p*-values compared to the null distribution. Thus, with *F*_*e *_denoting the empirical distribution, the ratio {1-*F*_*e*_(*η*)}/{1-*η*} will often be a good estimate of *p*_0 _for an astutely chosen threshold *η*. A spline is fitted to the function *p*_0_(*η*) = {1-*F*_*e*_(*η*)}/{1-*η*}, and the resulting function is evaluated at *η *= 1, yielding the estimate



(tilde '~' above *p*_0 _denoting the spline-smoothed version of *p*_0_(*η*)) which goes into the calculation of the FDR in (2), see Figure 15. Note the relationship with LSL, *p*_0_(*p*_(*i*)_) = (*N*-*i*)/{(*N*-*i*+1)*S*_*i*_*N*}. It can be shown that for fixed *η *this method offers a conservative point estimate of the FDR:

The q-value is estimated by combining (2), (7) and (11), and an implementation is provided as the function *qvalue *in package qvalue available on CRAN [18].

In [30] the authors go to great lengths to prove that for fixed *η*, as above, the conservativeness remains under various forms of dependency, such as clumpy dependence.

### The Bootstrap LSE

Another approach pioneered by Storey in [3] is to use a bootstrap least squares estimate (LSE), which chooses a value of *η *in *p*_0_(*η*), that minimises the variation of the estimate for random samples of the original *p*-values. The bootstrap standard reference [37] provides more theoretical background. Generate B new samples *p*-values p*^b ^(*b *= 1,..., *B*) by sampling with replacement from the observed ones, calculate a measure of the Mean Squared Error (MSE) of the corresponding estimates p*^b^_0_(*η*) for a lattice of values of *η *and choose the value minimising the MSE. More formally, the optimal η is obtained through



The version of the bootstrap used in this article uses more samples B than the version available in qvalue (B = 500 instead of B = 100), and seems to perform better (data not shown).

Available in functions *qvalue *[18] and *p0.mom *(in package SAGx) [18,38].

### SPLOSH

In [11] a spline function estimates the log-transformed pdf *log*[*f*(*x*)] using a complex algorithm involving splines called spacings LOESS histogram (SPLOSH). To obtain a stable estimate of FDR near zero a technique from mathematical analysis called l'Hospital's rule is used to approximate the ratio in (5) and to yield

,

where the numerator has been estimated as in (4). An R package with the same name is available [39].

The FDR estimate (6) is used with *F *obtained by the non-parametric estimate of the pdf.

Note that we can now calculate the posterior probability given its *p*-value that a gene is a DEG as *p*_1_(*x*) = 1 - *p*_0_/*f*(*x*), compare (3).

The method is available in R function *splosh *[40].

### BUM

In [10] the authors assume a beta-uniform (BUM) distribution, i.e. in (1) they replace *f*_1 _by a beta distribution,

*f*(*x*) = *λ *+ (1 - *λ*)*ax*^*a*-1 ^    (12)

where in addition to *λ *which corresponds to *p*_0 _the shape parameter *a *has to be estimated. Thanks to the simple form of the distribution it is possible to estimate parameters through the maximum likelihood principle, i.e. by choosing values that maximise the likelihood of observing the *p*-values that were actually observed. However, due to problem in identifying *p*_0 _with *λ*, the authors instead use an upper bound



which corresponds to *f*(1).

The FDR estimate (5) is used with *F *the cdf corresponding to (12).

The authors provide R code for the application of their method [39].

A more intricate Hierarchical Bayes model based on the beta-uniform concept allowing for different parameter values in different intervals appears in [41]. The R function localFDR provides an implementation of the method [42].

### Poisson regression

In [28] it is suggested to estimate any empirical distribution by dividing the real axis into intervals and regarding the number of hits in each interval as the result of an inhomogeneous Poisson process, much like counting the number of cars arriving at a crossing during different time intervals. This method was used in [27] to model the distribution of a transformed test statistic, it also appears in function *locfdr *which estimates a local FDR as a function of a test statistic. In our case, of course, the support of the distribution is the unit interval [0,1]. Then the expected number of hits in each subinterval of [0,1] can be modelled as a polynomial in the midpoints of the subintervals by a technique called Poisson regression (PRE). The approach taken here is to choose a polynomial of low degree so that the plateau representing the uniform distribution is well captured. In doing so the ability the capture the distribution at low *p*-values is sacrificed.

A more mathematical description now follows. The PRE method assumes that the counts *S*_*k *_follow a Poisson distribution whose intensity is determined by the midpoint *t*_*k *_of the interval *I*_*k*_, see [28]. To be specific: in the current application it is assumed that the expected frequency of observations in an interval is given by



where *μ*^*o*^_*k *_are the smoothed observed frequencies in each interval *I*_*k*_. In statistical jargon this is a Poisson regression model with *μ*^*o*^_*k *_as offset. This assumes independence between counts in different intervals. Although this does not hold true the model succeeds to capture the essential features of distributions. Standard functions in e.g. R can fit this model. Normalising the curve by the total number of *p*-values we get an estimate of the pdf. Finally, smooth the pdf *f*(*x*) with a spline to obtain a more stable result, and use the estimate (10). An implementation of PRE is provided through R function *p0.mom *in package SAGx [18,38].

### SEP

The Successive Elimination Procedure (SEP) excludes and includes *p*_*i *_successively such that the similarity of the distribution of the included tends to behave increasingly like a uniform [13]. Finally, an index set *J*_*final *_will map to a set of *p*-values that represent the true null hypotheses. This yields the point estimate



with *N*_*J *_= # *J*, the cardinality of the set *J*. The identification of *J*_*final *_proceeds by an intricate optimisation algorithm where the objective function consists of two terms : one Kolmogorov-Smirnov score



for the empirical cdf *F*_*J *_(based on *J*), to measure the distance to a uniform, and one penalty term to guard against overfitting



for some tuning parameter *λ*.

A local FDR is obtained from smoothed histogram estimates based on equidistant bins

,

where the function *h*_0 _refers to the *J*_*final *_set and *h *to the total set of *p*-values.

The function *twilight *in package *twilight *provides an implementation of SEP [19].

### Moment generating function approach

The next approach is based on the moment generating function (mgf), which is a transform of a random distribution, which yields a function *M*(*s*) characteristic of the distribution, cf. Fourier or Laplace transforms, e.g. [43]. Knowing the transform means knowing the distribution. It is defined as the expectation (or the true mean) of the antilog transform of *s *times a random variable *X*, i.e. the expectation of *e*^*sX*^or in mathematical notation:

*M*(*s*) = ∫*e*^*sx *^*f*(*x*)*dx*.

To calculate the mgf for p-values, we use the fact that the pdf is a mixture of pdf's (8). This yields the weighted sum of two transformed distributions:

,

where we have used the fact that the mgf of a uniform distribution over [0,1] equals *g*(*s*) = (*e*^*s *^- 1)/*s*. Denoting the second transform by *M*_1_(*s*) we finally have

*M*(*s*) = *p*_0_*g*(*s*) + (1 - *p*_0_)*M*_1_(*s*).     (13)

Now, the idea is to estimate these mgf's and to solve for *p*_0_. In the above equation *M*(*s*) can be estimated based on an observed vector of *p*-values and *g*(*s*) can be calculated exactly, respectively, while *p*_0 _and *M*_1_(*s*) cannot be estimated independently. The estimable transform is, given the observed *p*-values *p *= *p*_1_,..., *p*_*n*_, estimated by



Then, one can solve (13) for *p*_0_:



Let us do so for *s*_*n *_> *s*_*n*-1_, equate the two ratios defined by the right hand side in (14) and solve for *M*_1_(*s*_*n*_). This gives the recursion



**Figure 16:**
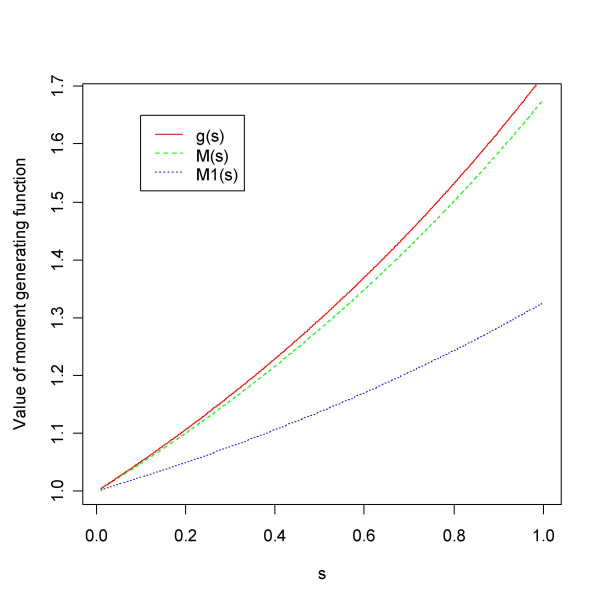
Estimated moment generating functions (mgf's). Given an observed vector of *p*-values it is possible to calculate mgf's for the observed distribution *f *(*M*) and the unobserved distribution *f*_1 _(*M*_1_), and without any observations we can calculate the mgf for the uniform (*g*).



If we can find a suitable start for this recursion we should be in a position to approximate the increasing function *M*_1_(*s*) for *s *= *s*_1 _<*s*_2 _< ... <*s*_*m *_in (0, 1]. Now, note that 1 ≤ *M*(*s*), for any mgf, with close to equality for small values of *s*. It makes sense to start the recursion with some *M*_1_(*s*_1_) in *I *= [1, *M*(*s*_1_)]. In general, it will hold true that 1 ≤ *M*_1_(*s*) <*M*(*s*) <*g*(*s*), since *f*_1 _puts weight to the lower range of the *p*-values at the expense of the higher range, the uniform puts equal weight, and *f *being a mixture lies somewhere in between. We can calculate *g*, *M *and *M*_1 _for an increasing series of values in [0,1], e.g. for *s *= (0.01, 0.0101, 0.0102, ..., 1). The output from one data set appears in Figure 16. Since all ratios (14) should be equal, a good choice of *M*_1_(*s*_1_) will be one that minimises the variation of the ratios. Standard one-dimensional optimisation will find the value in *I *that minimises the coefficient of variation (CV, standard deviation divided by mean)

where *s *= (*s*_1_, ..., *s*_*n*_). The CV will in contrast to the variance put both small and high values of the ratios on an equal footing and enable comparison.

Finally, these essentially equal ratios provide an estimate of *p*_0_.

A heuristic convexity argument suggests that mgf over-estimates *p*_0_, see the Additional file. Furthermore, the bias seems to decrease as *p*_0 _grows.

An implementation of mgf appears as function *p0.mom *in package SAGx [18,38].

### Local FDR and FDR

The concept of a local false discovery rate originates from [1]. Let the (true) local FDR at *t *be defined as the probability that gene *i *is unchanged conditional upon that its *p*-value equals *t*, or in formulas : *LFDR*(*t*) = *Pr*(*gene i unchanged *| *p*_*i *_= *t*) = *p*_0_/*f*(*t*). The Averaging Theorem of [4] states that integrating the local FDR over the rejection region *R*, such as *R *= [0, 0.01], yields the FDR : *FDR*(*R*) = *E*[*LFDR*(*y*) | *y *∈ *R*]. In [29] it is noted that the estimated q-value equals the mean of a local FDR



where the local FDR at the *i*^*th *^ordered *p*-value *p*_(*i*) _equals

,

where N denotes the total number of genes. This rephrases the theorem in terms of estimators. The local FDR is meant as an approximation of the probability that an individual gene *i *is a DEG. As remarked in [29] the q-value does not estimate the probability that a gene is a false positive. Indeed, the theorem shows that it is the mean of that probability for all genes at least as extreme as *i*. Thus the q-value will tend to give a lower value than *LFDR*(*i*).

Under a wide range of models, where *f*(*x*) is non-increasing, e.g. the BUM model, the expected local *LFDR*(*i*) will be non-increasing, and hence the differences above should tend to increase, see the Additional file. Hence there is a need for enforcing monotonicity as in (7). One tool for enforcing monotonicity is the Pooling of Adjacent Violators (PAVA) algorithm [44]. This algorithm has an intuitive appeal, is less ad-hoc than the local spline approach presented in [29], and is the non-parametric maximum likelihood estimate under the assumption of monotonicity. As an example of how it works, consider the series (1,2,4,3,5), which PAVA turns into the non-decreasing series (1, 2, 3.5, 3.5, 5) by pooling the violators of monotonicity (4, 3) and replacing them by their mean. Though not equivalent to the q-value from (2) and (7), the results from applying PAVA to the terms in (15) agreed rather well with the values obtained from function *qvalue*. In the **Results **section this approach combined with the PRE estimate of *p*_0 _is referred to as pava FDR. We could have used mgf for calculating FDR, but it was excluded due to the better over-all performance of PRE.

The bootstrap LSE gives a very similar result to the smoother and thus was excluded in comparison of FDR estimates.

The R function *pava.fdr *in package SAGx provides an implementation of pava FDR, and returns a list of estimates of FDR, LFDR and *p*_0 _[18,38].

The reference [25] presents the theory behind the estimation of local false discovery rates provided by R package *locfdr *[18,25]. The method procedes by transforming the test statistic *t *into *Z *= Φ^-1^(*m*(*t*)), where Φ is the cdf corresponding to N(0,1) and *m *is a transform that for the uninteresting/null class renders the distribution of Z into a N(0,1). Typically, *m *could equal the cdf of the *t*-distribution. Assuming the model (1) the method estimates the mixture density *f*^*t *^using Poisson regression, and fits either a theoretical null sub density (from now on suppressing superscript *t *and denoting the pdf corresponding to Φ by *φ*) *f*^+^_0_(*z*) = *p*_0 _*φ*(*z*) around z = 0, or fits an empirical null distribution. Then the procedure estimates the local false discovery rate *fdr*(*z*) = *f*^+^_0_(*z*)/*f*(*z*).

**Figure 3:**
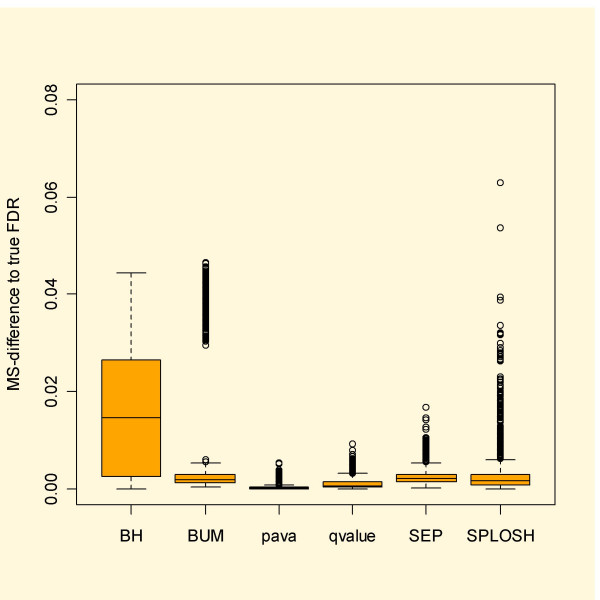
Mean squared difference between estimated and true FDR for the simulated independent data. Note that qvalue and pava FDR stand out for having the smallest deviation from the true FDR.

## Supplementary Material

Additional File 1 contains further results concerning weakly dependent data and smaller datasets, as well as some mathematical details supporting points made in the article. Click here for file
