# Supplementary material for: A comparative review of estimates of the proportion unchanged genes and the false discovery rate
Source: BMC Bioinformatics. 2005 Aug 8;6:199. doi: 10.1186/1471-2105-6-199 (PMC1199583; doi:10.1186/1471-2105-6-199)
Supplement: Additional File 1 — contains further results concerning weakly dependent data and smaller datasets, as well as some mathematical details supporting points made in the article. [file 1471-2105-6-199-S1.doc]

# Additional file

## Weakly dependent data

Data were also simulated using the clumpy dependency protocol but with the block-wise sample term following a N(0, 1/20) distribution. This simulates a situation with much less dependency between genes, and the outcome reminds a great deal of the independent case, see Figures A1,2.

**Figure A1**. Estimation of *p_0_* with weakly dependent data. The results resemble those for the independent case, except when it comes to BH, whose overestimation slumps and transforms into underestimation in some cases.

**Figure A2**. Estimation of FDR with weakly dependent data.

**Figure A3**. Estimation of *p_0_* in small dataset with weak dependency. The clumpy dependence protocol was used to generate the expression of 300 genes. All methods exhibit great difficulties with these data.

**Figure A4**. Estimation of FDR in a small dataset with weak dependency.

**Figure A5.** Simulated weakly dependent data corresponding to 5000 genes.

**Figure A6.** Simulated weakly dependent data corresponding to 5000 genes.

## A

The differences between consecutive ordered *p*-values *p_(i)_ – p_(i-1)_* will tend to increase, if the p-value distribution has a continuous pdf *f* and is non-increasing. Let the distribution function be *F* , and let *Q_i/n_* the number fulfilling *F(Q_i/n_) = i/n*. Then, assuming *n* large *E[p_(i)_] ≈ F(Q_i/n_)*, see [1]. Hence,

$$E\left[p_{\left(i\right)}-p_{\left(i-1\right)}\right]=\int_{Q_{\left(i-1\right)/n}}^{Q_{i/n}} f\left(x\right)dx=\left(Q_{i/n}-Q_{\left(i-1\right)/n}\right)f\left(x_{0}\right)$$

where in the second equality the mean value theorem was invoked for some *x_0_* in the interval *(Q_(i-1)/n_, Q_i/n_).* By definition the integral equals 1/n. Since *f* increases when the argument approaches 0, the difference *p_(i)_ – p_(i-1)_* must in expectation decrease in proportion to keep the product fixed at *1/n*.

## B

The ratio of unbiased estimates of the numerator and denominator in (3) will tend to over-estimate the true FDR. Denote by *R* the number of rejected and by *V* the number of false positives, given some cut-off *α*. This follows from the Gauss approximation formula applied to the ratio of two random variables (using the notation *μ_X_ = E[X]* )

$E\left[\frac{V}{R}\right]=\frac{\mu_{V}}{\mu_{R}}+\sigma_{R}^{2}\frac{\mu_{V}}{\mu_{R}^{3}}-\frac{Cov\left(R,V\right)}{\mu_{_{R}}^{2}}$.

Now assume genes to be independent. Then *Cov(R, V) = Cov(V+T,T) = σ_V_^2^*, so that the right hand side above becomes

$\frac{\mu_{V}}{\mu_{R}}+\frac{p_{0}N^{2}\alpha F\left(\alpha\right)\left(1-F\left(\alpha\right)\right)}{\left(NF\left(\alpha\right)\right)^{3}}-\frac{p_{0}N\alpha\left(1-\alpha\right)}{\left(NF\left(\alpha\right)\right)^{2}}=\frac{\mu_{V}}{\mu_{R}}+\frac{p_{0}\alpha\left(\alpha-F\left(\alpha\right)\right)}{NF\left(\alpha\right)}$,

where *F* is the distribution function of *p*-values.

Since typically *F(α) ≥ α*,

$E\left[\frac{V}{R}\right]\leq\frac{E\left[V\right]}{E\left[R\right]}$,

with close to equality as *N* increases. To extend the result to dependent genes, one would have assume some dependence structure as in [2].

## C

The following heuristic indicates that estimates based on (14) will tend to overestimate. From now on drop the argument *s* in the mgf’s. By the Central Limit Theorem [3] *M* and *M_1_* will closely follow a bivariate normal distribution. If we condition on the unobservable *M_1_* and use normality, then

Since this ratio will be a convex function in the estimate of *M_1_* for permitted values of *p_0_*, *M*, *g* and *M_1_*, its expectation is by Jensen’s inequality bounded from below by *(M – M_1_)/(g – M_1_) = p_0_*. Note that this assumes that our estimate of *R_1_* is unbiased or underestimates the true value.

## References

1. Cox D, Hinkley D: **Theoretical Statistics**: Chapman & Hall; 1974.

2. Storey J, Tibshirani R: **Estimating false discovery rates under dependence, with applications to DNA microarrays**. In: *Technical Report 2001-28, Department of Statistics, Stanford University.* 2001.

3. Feller W: **An Introduction to Probability Theory and Its Applications**, vol. 2, Second edn. New York: John Wiley; 1971.
